# Successful experimental infant baboon model for childhood cryptosporidiosis studies

**DOI:** 10.1186/s13071-021-04804-4

**Published:** 2021-06-10

**Authors:** Ngalla E. Jillani, Atunga Nyachieo, Daniel C. Chai, James Nyabuga Nyariki

**Affiliations:** 1 Institute of Primate Research, Box 24481-00502, Karen Nairobi, Kenya; 2 Department of Biochemistry and Biotechnology, Technical University of Kenya, P.O. Box 52428-00200, Nairobi, Kenya

**Keywords:** Cryptosporidiosis, Infant baboon, *Cryptosporidium parvum*, Children

## Abstract

**Background:**

Cryptosporidiosis causes high morbidity and mortality in children under 2 years of age globally. The lack of an appropriate animal model that mimics the pathogenesis of disease in humans has hampered the development and testing of potential therapeutic options. This study aimed to develop and validate an infant baboon infection model of cryptosporidiosis.

**Methods:**

Eighteen immunocompetent weaned infant baboons aged 12 to 16 months were used. The animals were *n* = 3 controls and three experimental groups of *n* = 5 animals each inoculated with *Cryptosporidium parvum* oocysts as follows: group 1: 2 × 10^4^, group 2: 2 × 10^5^, group 3: 2 × 10^6^ followed by daily fecal sampling for oocyst evaluation. Blood sampling for immunological assay was done on the day of infection and weekly thereafter until the end of the experiment, followed by necropsy and histopathology. Statistical analysis was performed using R, SPSS, and GraphPad Prism software. Analysis of variance (ANOVA) and Bonferroni post hoc tests were used for comparison of the means, with *p* < 0.05 considered as a significant difference. Correlation coefficient and probit analysis were also performed.

**Results:**

In all experimental animals but not controls, the onset of oocyst shedding occurred between days 2 and 4, with the highest oocyst shedding occurring between days 6 and 28. Histological analysis revealed parasite establishment only in infected animals. Levels of cytokines (TNF-α, IFN-γ, and IL-10) increased significantly in experimental groups compared to controls.

**Conclusion:**

For developing a reproducible infant baboon model, 2 × 10^4^ oocysts were an effective minimum quantifiable experimental infection dose.

**Graphic abstract:**

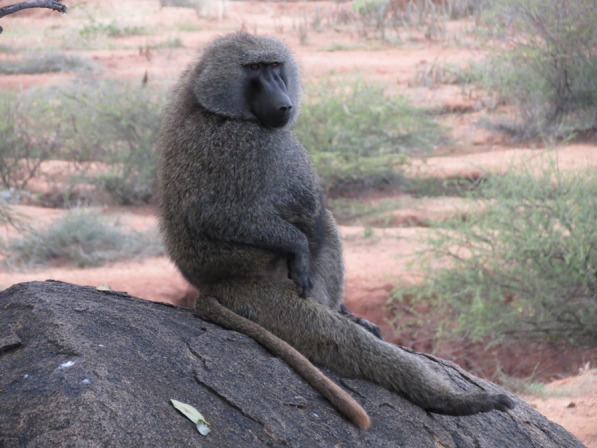

## Background

Cryptosporidiosis is an infectious disease caused by an apicomplexan oocyst-forming protozoan parasite, *Cryptosporidium* spp. [1, 2]. Besides the two main species of *Cryptosporidium* infecting humans, namely *C. hominis* (which infects humans only) and *C. parvum* (which infects both humans and bovines), other species such as *C. suis*, *C. meleagridis*, *C. andersoni*, *C. felis*, *C. canis*, *C. muris*, and *C. baylei* occasionally infect people as well [2, 3]. This enteropathogenic disease is associated with diarrhea in humans of all ages, but more severe in children under 2 years worldwide [4, 5]. This neglected zoonotic disease, with a typically underestimated prevalence of 2–12% in the developing world, has received little research attention, resulting in a limited understanding of its pathogenesis and less effective therapeutic solutions [3, 6]. Childhood cryptosporidiosis presents a huge disease burden in the developing world, causing morbidity and mortality, with possible long-term learning deficits, stunted growth, and impaired physical fitness in affected children [2, 6–8].

Transmission is by the fecal–oral route through direct contact with infected humans, animals, contaminated drinking water, or agricultural products [9]. Oocysts excreted in feces can remain infectious for up to 2 months [10]. This disease causes opportunistic infections in both immunocompetent and immunocompromised persons [5]. While the incidence of cryptosporidiosis varies throughout the year, high levels occur during the warm rainy months [8].

Despite numerous attempts to control *Cryptosporidium* infections, there have not been effective animal models that accurately mimic the disease in humans, as tools for testing newly developed or repurposed molecules for potential drugs that could effectively treat this condition [2, 7]. To better understand the disease, various animal models, mainly murine and porcine models, have been utilized [11]. The findings from these lower animal models, however, cannot be extrapolated to humans, since the disease pattern does not mirror that in humans due to the huge phylogenetic gap. Non-human primates, which are phylogenetically closer to humans, are therefore attractive as models for understanding this disease [7]. Indeed, previous studies in pigtailed macaques demonstrated that these non-human primates were good models of the disease [7]. Similarly, in another study involving a cross-sectional survey in captive baboons [12], *C. hominis* infection was detected mostly in infant baboons, confirming that baboon infants are susceptible to *Cryptosporidium hominis* infection and could be useful in the development of a reproducible experimental infection model of childhood cryptosporidiosis. However, there is no study showing dose-controlled *Cryptosporidium* infection in baboon infants. Thus, the main objective of this study was to validate and develop a reproducible infant baboon infection model of childhood cryptosporidiosis through a quantifiable *Cryptosporidium* dosage using *Cryptosporidium parvum*.

## Methods

### Selection of animals

Weaned immunocompetent infant baboons (*n* = 18) of both sexes (10 males and 8 females) aged between 12 and 16 months and weighing between 3.0 and 7.1 kg were recruited into the study. The sample size (*n*) was calculated using the power analysis equation *n* = 1 + 2*C*(*s*/*d*)^2^. The animals were screened and treated for enteric worms and diarrhea-causing bacteria, namely *Shigella* spp. and *Salmonella* spp., before inoculation with doses of *Cryptosporidium parvum* oocysts. They were then housed in cages that meet international standards in size and space based on the NIH *Guide for the Care and Use of Laboratory Animals*, eighth edition. Animals were fed commercial chow made by Unga Group Limited, based on our formulation, while water was given ad libitum. They were given fruits three times a week and provided with enrichment devices, which were changed weekly.

### Isolation of *Cryptosporidium* oocysts

*Cryptosporidium parvum* oocysts used in this study were isolated by the floatation method and identified by acid-fast staining (modified Ziehl–Neelsen method) as described previously [7]. In this method, a lump of the fecal sample (about 1 g) was put into a container and 5 ml of distilled water was added, thoroughly mixed, then sieved into a beaker. The filtrate was then transferred into a 15 ml centrifuge tube and topped up with distilled water to 15 ml, then centrifuged at 1500 rpm for 10 min. The supernatant was discarded, and 5 ml of sucrose solution of specific gravity 1.27 was added and vortexed for 1 min. More sucrose solution was added to bring it to 15 ml, until a convex meniscus was formed. It was then covered with a coverslip and centrifuged at 1500 rpm for 10 min. The coverslip was then carefully removed and washed by sprinkling PBS on it into a glass beaker. The 15 ml tube was again topped up with sucrose solution until it formed a meniscus, covered with a coverslip, centrifuged for another 10 min at 1500 rpm, and oocysts were recovered as described earlier. This process was repeated three times to ensure the maximum recovery of oocysts. The resultant solution with recovered oocysts from the beaker was then put into a 15 ml tube, topped up with PBS, and centrifuged at 1500 rpm for 10 min. The supernatant was discarded and the resultant pellets were suspended in 1.5 ml PBS. One microliter of this solution was examined under a microscope to determine the number of oocysts in a 1 μl volume. This volume estimation for oocyst numbers was then used to constitute appropriate inoculation doses.

### Inoculation of animals

All selected experimental animals (*n* = 18) were starved in the morning of inoculation, and the procedures were carried out under general anesthesia. This involved the administration of ketamine HCl at 10 mg/kg body weight mixed with xylazine 5%, injected intramuscularly. During this time, blood samples, temperature, and body weight measurements were taken. The animals were randomized to three experimental groups using sealed opaque envelopes. Quantified parasite doses were administered to the animals on the table according to the groups. The doses comprised three concentrations of the parasite: group 1: 2 × 10^4^ (*n* = 5), group 2: 2 × 10^5^ (*n* = 5), and group 3: 2 × 10^6^ (*n* = 5). Group 4 comprised control animals (*n* = 3), which were given normal saline. Parasite dose administration was done through a thin tube pushed into the orogastric route and the parasite's oocysts delivered through a syringe connected to the tube and washed several times into the gastrointestinal tract. The animals were then returned to their cages and closely monitored until they recovered from the effects of anesthesia.

### Stool sampling and oocyst quantification

Stool sampling for quantification of shed oocysts was done daily by placing a tray at the base of the cage, just below the animal. Part of freshly voided fecal matter was put in sealable vials and stored at four degrees (4 °C). Later, 100 g of each sampled stool was concentrated by formalin-ethyl acetate sedimentation and stained using the modified Ziehl–Neelsen method [7]. A 5 μl volume of the isolated oocysts was sampled, counted under a microscope (×400) and total numbers recorded.

### DNA extraction from oocysts

Chromosomal DNA was extracted using the QIAamp DNA kit (Qiagen, Hilden, Germany) according to the manufacturer’s instructions. Five microliters of pure oocysts was transferred into an Eppendorf tube and dissolved in 700 μl of ASL buffer of the DNA extraction kit. This was then exposed to five freeze/thaw cycles in liquid nitrogen and boiling water to disrupt oocyst walls. Afterward, 700 μl of ASL buffer was added into the sample tube and then the procedure, based on the instruction of the DNA extraction kit was followed.

### Nested PCR amplification

PCR was carried out using a peqSTAR thermocycler (Peqlab, Erlangen, Germany) as described previously [13]. Briefly, 18S ribosomal RNA gene from *Cryptosporidium* genus (NCBI, Accession No. GQ259149.1) was amplified under the following conditions for analysis: 94 °C for 5 min, 35 cycles of 1 min at 94 °C, 1.30 min at 60 °C, 2 min at 72 °C (Cry18S-S2, 5′ GGTGACTCATAATAACTTTACGG 3′ as forward and Cry18S-As2, 5′ ACGCTATTGGAGCTGGAATTAC 3′ as reverse primers). This was followed by nested amplification of 35 cycles: 1 min at 94 °C, 1.30 min at 60 °C, 2 min at 72 °C and a final extension step of 10 min at 72 °C (Cry18S-S1, 5′ TAAACGGTAGGGTATTGGCCT 3′ as forward and Cry18S-As1, 5′ CAGACTTGCCCTCCAATTGATA3′ as the reverse. Aliquots of 20 ρM of each primer were added in a volume of 50 μl containing 20 mM (NH4)2 SO4, 75 mM Tris–HCl (pH. 8.8), 1 mM MgCl2, 0.2 mM dNTP mix, 1.2 units of thermostable DNA polymerase (Invitrogen, Mannheim, Germany), and 1 μl of the template (genomic DNA). The amplification products were subjected to electrophoresis on 1% agarose gel stained with ethidium bromide and visualized under a UV transilluminator. The expected PCR band size after nested PCR was approximately 240 bp.

### Sequencing

The PCR bands were extracted from the agarose gel and purified then sequenced by Macrogen Co. Ltd. (Netherlands). The sequenced PCR fragment was then blasted at NCBI (https://blast.ncbi.nlm.nih.gov/Blast.cgi) to identify the sequenced gene.

### Blood sampling

Six milliliters of whole blood was collected from each animal on inoculation day and 7 days thereafter for processing blood cell count and cytokine assays. Blood was collected from femoral vein under anesthesia attained by intramuscular injection of a ketamine-xylazine mixture with ketamine HCl at a dosage of 10 mg/kg body weight and xylazine 5%. While 3 ml of the collected blood was processed for cell count, the other 3 ml were processed for serum, which was then stored at −20 °C. Cell counting was done using an automated differential cell counter (Coulter Ac·T 5diff CP, Beckman Coulter, Miami, FL, USA). Serum was separated from whole blood through centrifugation using a Jouan C422 centrifuge (LabCare America, FL, USA) at 1500×*g* for 10 min and then collected into a clean tube for storage.

### Temperature and body weight

Body temperatures were recorded using a rectal thermometer (Hongwei Technology Co. Ltd, Hong Kong) inserted into the anal opening, while body weights were taken on a weighing balance. These were taken during the day of inoculation and weekly thereafter for the duration of the experiment.

### Immunological analysis

From the serum of the collected blood samples, inflammatory cytokines TNF-α, IFN-γ, and IL-10 were analyzed using ELISA kits (human TNF-α kit, IFN-γ kit, and IL-10 kit; Thermo Fisher Scientific Ltd, Vienna, Austria) at acute phase day 14 and chronic phase day 49 for both control and experimental animals. A sandwich ELISA protocol was employed for detecting the presence and levels of the inflammatory cytokines TNF-α, IFN-γ, and IL-10. Briefly, Corning Costar 9018 low-bottom high binding ELISA plates (Thermo Fisher Scientific Ltd, Vienna, Austria) were coated with 100 μl of capture antibody. The plates were then sealed and incubated overnight at 4 °C. This was followed by washing the plates three times with washing buffer. After the last wash the plates were blocked with 100 μl of blocking buffer and incubated for 2 h at room temperature, then washed three times using washing buffer. Specific standards for TNF-α, IFN-γ, and IL-10 were diluted twofold with the 1× assay diluent. One hundred microliters of appropriate standard or 50 μl of serum (probe) were added to separate wells followed by incubation for 2 h at room temperature on a shaker. The plates were thereafter washed five times. The respective detecting antibodies were diluted with 1× assay diluent then 100 µl of this added into each well followed by incubation for one hour at room temperature while shaking. Thereafter the plates were washed five times and then 100 μl of streptavidin-peroxidase for IFN-γ (1:200) and avidin-HRP for IL-10 and TNF-α (1:200) were added into each well in appropriate plates followed by incubation for 30 min at room temperature. The wells were washed three times using washing buffer. One hundred microliters of TMB was added in each well then incubated for 15 min in the dark. Thereafter, 50 μl of the stop solution (2 N H_2_SO_4_) was added to all the wells. The plates were then read using an ELISA reader at 450 nm wavelength.

### Postmortem and histological analysis

At the end of the experiment, animals were humanely sacrificed and tissues harvested for histological analysis. Tissues from the small intestine (ileum) and caecum were processed, sectioned at 4 µm using a rotary microtome (KD 2258, TED PELLA, Inc., CA, USA), and subjected to hematoxylin–eosin staining followed by microscopic analysis.

### Statistical analysis

Various statistical software programs were used for statistical analysis: R Statistics version 3.5.1 (The R Foundation, Vienna, Austria); SPSS version 25 statistical software (IBM SPSS, NY, USA), and GraphPad Prism 5.0 (GraphPad Software, San Diego, CA, USA). ANOVA and Bonferroni post hoc tests were used for comparison of the mean number of oocysts shed and cytokine levels. Levels of significance were set at 95% CI with *p* < 0.05 considered a significant difference. The correlation coefficient (R-values) was used to compare the relationship between the number of oocysts shed and the lymphocytes produced during the establishment of an infection. Probit analysis was performed for the prediction of minimum dosage capable of causing an infection, using SPSS version 25 statistical software (IBM SPSS, NY, USA).

## Results

**Fig. 1:**
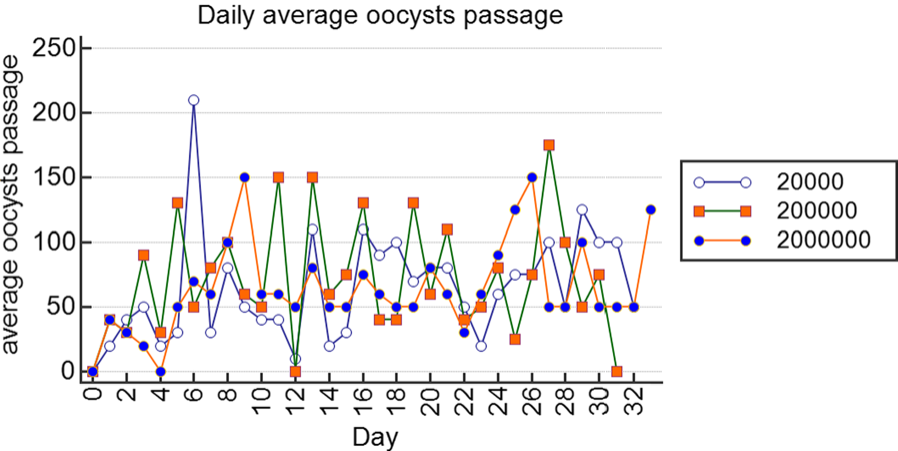
Plots of daily average oocyst shedding for the different doses using R Statistics version 3.5.1. In all experimental animals, the onset of oocyst shedding occurred between days 2 and 4 and continued throughout the experimental period, with the highest oocyst shedding occurring between days 6 and 28, indicating that the acute phase of the experimental disease occurred between days 6 and 28, while the chronic phase occurred from day 30 to the end of the experiment
  (iii) *Oocyst shedding patterns between males and females*There were no significant differences between oocyst shedding patterns in males and females throughout the experimental period within and between infected groups (*p* > 0.05).

- Clinical parameters
  (i) *Stool texture, body weight, and temperature changes.* Stool texture for experimental animals was either loose (stools spread on a surface), poorly formed (stools shaped up when shed but easily spread to touch), or well-formed (hard stools that remained firm to touch), with a few animals showing watery stools (fluid stools that run on a surface). All control animals produced well-formed stools. This study showed that there were no significant differences in temperature or body weight changes between or within the experimental groups and control groups (*p* > 0.05).
  (ii) *Oocyst shedding and infection doses*. In all experimental animals (oocysts doses: group 1; 2 × 10^4^, group 2; 2 × 10^5^, group 3; 2 × 10^6^) but not controls, the onset of oocyst shedding occurred between days 2 and 4 (mean 36.7 ± 8.82, 50.0 ± 20.0, 25.0 ± 5.0; *p* = 0.56; respectively; Fig. 1) and continued throughout the experimental period, with the highest oocyst shedding consistently occurring between days 6 and 28 (mean 69.5 ± 9.3; 78.6 ± 9.7; 73.2 ± 6.7; *p* = 0.82, respectively). By day 30, the number of oocysts shed averaged to a constant of between 50 and 100 up to the end of the experiment. Thus, the acute phase of the experimental disease occurred between days 6 and 28, while the chronic phase began on day 30 and continued up to day 49 of the experiment.

**Fig. 2:**
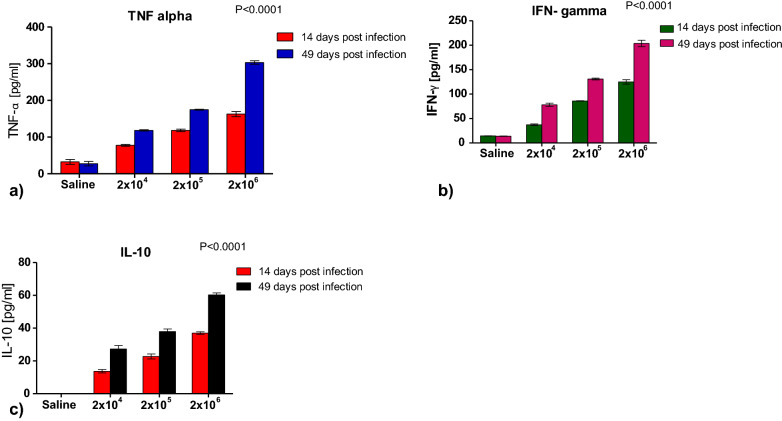
Comparison of levels of pro-inflammatory and anti-inflammatory cytokines between experimental and control animals. **a–c** Showing levels of pro-inflammatory cytokines (TNF-α, IFN-γ) and anti-inflammatory cytokines (IL-10) in experimental groups compared to controls on day 14 and 49 post infection using GraphPad Prism 5.0. **a** Pro-inflammatory cytokine TNF-alpha increased (almost twofold) on day 14 as compared to day 49 in the experimental groups as parasite dosage increased. **b** Pro-inflammatory cytokine IFN gamma increased (almost twofold) on day 14 as compared to day 49 in the experimental groups as parasite dosage increased. **c** Anti-inflammatory cytokine IL-10 increased (almost twofold) on day 14 as compared to day 49 in the experimental groups as parasite dosage increased

**Fig. 3:**
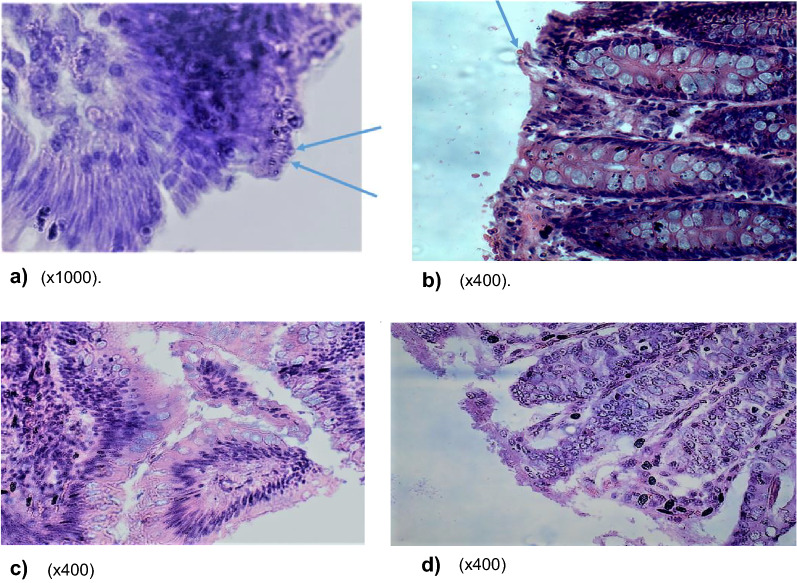
**a**–**d** Comparison of parasite establishment within ileum and caecum epithelia of experimental and control animals. **a**–**d** Demonstrates parasite establishment in a vacuole within the epithelia of ileum and caecum in only experimental animals and not the controls. Both **a** and **b** show the establishment of the parasite in the epithelial mucosa of the ileum and caecum in experimental animals, while **c** and **d** (controls) show normal ileum and caecum mucosa without parasite infection. **a** Photomicrograph of ileum section with oocysts embedded in mucosa lining (×1000). **b** Photomicrograph of caecum section with oocysts embedded in mucosa lining (×400). **c** Photomicrograph showing no oocysts in mucosa of normal ileum epithelia in controls (×400). **d** Photomicrograph showing no oocysts in mucosa of normal caecum epithelia in controls (×400)
- *Molecular typing of oocysts.* Molecular analysis (PCR followed by sequencing and alignment of the data) confirmed that oocysts used to infect the experimental animals in this study were those of *Cryptosporidium parvum.*
- *Lymphocyte levels.* A comparison of mean absolute lymphocytes between experimental animals and controls, especially during infection establishment (days 2–4), the acute phase (days 6–28) and the chronic phase (days > 30), confirmed that lymphocytes increased in all experimental groups but not in the controls with a positive correlation between the lymphocytes and the number of oocysts shed on days 0, 7, 14, 21 and 28 for all the experimental groups (group 1, *r* = 0.82; group 2, *r* = 0.48; group 3, *r* = 0.43).
- *Immunological data (cytokines).* Immunoassays revealed a significant increase in levels of pro-inflammatory cytokines (TNF-α, IFN-γ) and anti-inflammatory cytokines (IL-10) in experimental groups compared to controls on day 14 and 49 post-infection (*p* < 0.01) (Fig. 2a–c). This increase in inflammatory cytokines corresponded to an increase in the parasite dosage used for infection. It became evident that among the experimental groups, the levels of these cytokines were higher on day 49 compared to levels on day 14 post-infection (almost twofold). The observed significant increase in pro-inflammatory cytokines TNF-α and IFN-γ corresponded to an upsurge in anti-inflammatory cytokine, IL-10 as parasite dosage increased in the experimental groups (Fig. 2a–c).
- *Postmortem and histopathological data.* Postmortem results showed signs of possible parasite infection only in experimental animals and not in the controls. These included swollen mesenteric lymph nodes and spleen follicular hyperplasia; enlarged adrenal glands; blood vessel congestion in the colon, ileum, kidney, and brain; vanish gray hepatization with multiple focal fibrosis in the liver, a case of suspected hepatocystis. The possibility of parasite (*C. parvum*) infection was indeed confirmed by histological data which revealed parasite establishment in the ileum and caecum epithelia in experimental animals but not in the controls (Fig. 3a–d).
- *Prediction model for minimum infective dosage.* Using our data, the minimum hypothetical predictive infective dose (ID) for 50% (ID_50_) required to cause an infection, was calculated through a probit statistical model and found to be 25 oocysts.

## Discussion

This study established a reproducible infant baboon model for childhood cryptosporidiosis studies using isolates of *Cryptosporidium parvum*. Various parameters supporting this model were evaluated including oocyst shedding, body weight and temperature changes, stool texture, lymphocytes, cytokines, postmortem observations, and histopathology. Herein, we compare our findings with those in humans, rhesus monkeys, and mice, which further confirms our model.

Clinical observation showed that stool texture for our experimental animals was either loose, poorly formed, or well-formed, with a few animals showing watery stools. All control animals produced well-formed stools. Similar observations exist in human and rhesus monkeys, where diarrhea was an indication but did not necessarily predict infection [14]. These findings contrast with those in mice, which do not show any clinical signs [7, 15].

In this study, there were no significant differences in temperature or body weight changes between or within the experimental groups, as observed in humans and rhesus monkey studies [7, 14].

In our study, all three different dosages established an infection, indicating that a lower dosage of 2 × 10^4^ oocysts was as effective as higher doses of 2 × 10^5^ and 2 × 10^6^. This low infective dosage was lower than the 2 × 10^5^ reported in rhesus monkeys [7]. Though not done in this study, it is possible that an even lower dosage of < 2 × 10^4^ oocysts could also have established infection. Indeed, the minimum hypothetical infective dose (ID) for 50% (ID_50_) required to cause an infection in a study, derived from a Probit statistical model, is 25 oocysts, which is closer to the 10–50 oocysts reported in rhesus monkeys [7] but far less than the LD_50_ of 79 oocysts for mice [15].

As to whether the risk of infection is gender-based, it was clearly demonstrated that oocyst shedding patterns were similar between male and female baboons within and between infection groups. This finding has been reported consistently in humans, where the risk of contracting *Cryptosporidium* was insignificant between sexes [14], but such comparisons had not been demonstrated in rhesus monkeys [7].

The onset of oocyst shedding in all experimental animals but not controls between days 2 and 4, with the highest oocyst shedding occurring between days 6 and 28, considered as the acute phase of the disease in this study, is similar to observations in humans and rhesus monkey studies, where infection is established in 3–7 days and oocyst shedding continues for 3–4 weeks independent of the dosage [7, 16–18]. However, this is in contrast to mice, in which oocyst shedding occurs on day 6 post-inoculation and lasts for only 5–12 days [5, 19].

As expected, experimental animals, and not the controls, showed oocyst embedding onto the epithelial mucosa of both the caecum and ileum, as demonstrated in other studies in mice and humans, where oocysts were observed to embed onto the epithelial lining of the duodenum, jejunum and ileum [20, 21].

Analysis of lymphocytes indicated a positive correlation with the number of oocysts shed as shown on days 0, 7, 14, 21, and 28 for all the experimental groups. The increase in the number of lymphocytes relative to the increase in shed oocysts indicated a successful *Cryptosporidium* infection. This is similar to humans but has not been evaluated in rhesus monkeys and mice [16, 17].

The pro-inflammatory cytokines TNF-α and IFN-γ are good indicators of the establishment of parasite infection in most apicomplexan protozoan infections, similar to what would be observed in *Plasmodium* spp. in malaria and *Toxoplasma gondii* in toxoplasmosis [22]. The significant increase in pro-inflammatory cytokines TNF-α and IFN-γ observed in our current study suggests a Th1 immune response at play. This upregulation of TNF-α and IFN-γ can be interpreted as either enhancing protection against *Cryptosporidium* or rather causing tissue inflammation and is corroborated by tissue pathology from histology sections, which demonstrated the presence of parasite on the epithelium of both ileum and caecum of only experimental animals but not the controls. The observed rise in IL-10 levels is indicative of the body’s attempt at regulating the pro-inflammatory cytokines to bring about the balance of interplay between innate and adaptive immune response. Furthermore, elevation in levels of cytokines (TNF-α, IFN-γ, and IL-10) in experimental animals but not in the control animals corresponds to a study on humans [23] and also corroborates the histological analyses, confirming the establishment of *Cryptosporidium* infection in the experimental animals and not the controls. Indeed, the increase in these cytokines also corresponded to an increase in the number of *Cryptosporidium* oocysts shed in the experimental animals.

In addition, the pathologies observed in experimental animals during postmortem results in our study indicated tissue inflammation/swelling, which may be partly explained by immune reactions elicited by the infection [24, 25] as well as possible modulated immunity due to gut microbiome imbalance possibly resulting from the presence of *Cryptosporidium* in the gut. All these may have contributed to a cytokine storm elicited by the infection, leading to the observed global organ pathologies [26].

## Conclusions

The series of experiments conducted in this study resulted in the establishment of a reproducible infant baboon infection model with an effective minimum infection dose of 2 × 10^4^ oocysts of *Cryptosporidium parvum*. This experimental model is a useful tool for studying the pathogenesis of childhood cryptosporidiosis.

## Data Availability

The datasets used and/or analyzed during the current study are available from the corresponding author on reasonable request.

## Abbreviations

PBS: Phosphate-buffered saline
DNA: Deoxyribonucleic acid
PCR: Polymerase chain reaction
ASL: Adenylosuccinate lyase
RNA: Ribonucleic acid
NCBI: National Center for Biotechnology Information
UV: Ultraviolet
bp: Base pairs
ELISA: Enzyme-linked immunosorbent assay
TMB: Tetramethylbenzidine
ANOVA: Analysis of variance
SPSS: Statistical Package for the Social Sciences
TNF-α: Tumor necrosis factor-alpha
IFN-γ: Interferon gamma
IL-10: Interleukin-10
